# Scalable biomimetic SARS-CoV‑2 nanovaccines with robust protective immune responses

**DOI:** 10.1038/s41392-022-00942-y

**Published:** 2022-03-25

**Authors:** Xuenian Chen, Tongfei Shi, Chao Yang, Fangman Chen, Xuan He, Kunbao Zhang, Hanze Hu, Lulu Cai, Kam W. Leong, Dan Shao

**Affiliations:** 1 School of Biomedical Sciences and Engineering, South China University of Technology, Guangzhou International Campus, Guangzhou, Guangdong 511442 China; 2 Institutes of Life Sciences, School of Medicine, South China University of Technology, Guangzhou, Guangdong 510006 China; 3 National Engineering Research Center for Tissue Restoration and Reconstruction, South China University of Technology, Guangzhou, Guangdong 510006 China; 4 Department of Biomedical Engineering, Columbia University, New York, NY 10027 USA; 5 Personalized Drug Therapy Key Laboratory of Sichuan Province, Department of Pharmacy, Sichuan Provincial People’s Hospital, School of Medicine, University of Electronic Science and Technology of China, Chengdu, 610072 China; 6 Guangdong Provincial Key Laboratory of Biomedical Engineering, South China University of Technology, Guangzhou, 510006 China; 7 Key Laboratory of Biomedical Material and Engineering of the Ministry of Education, South China University of Technology, Guangzhou, 510006 China

**Keywords:** Nanobiotechnology, Infectious diseases

**Dear Editor**,

The emergence of the severe acute respiratory syndrome coronavirus 2 (SARS-CoV-2) infection requires rapid development of vaccines matching the pace of virus mutation. While the first-generation of nucleic acid vaccines have been successful, subunit vaccines carry far fewer safety concerns and also have shown promise in clinical trials.^1^ Innovations in biomaterials science and nanotechnology have produced nanoparticulate subunit vaccines that can target the Receptor-Binding Domain (RBD) of the spike protein to generate a robust immune response for immunization.^2,3^ However, the time-consuming and costly recombinant SARS-CoV-2 RBD production, together with challenging quality control, diminish the appeal of adopting this approach for developing vaccine candidates for mass immunization. A vaccine design that is more economical, versatile, and manufacturable would be needed to combat the evolving COVID-19 pandemic.

Enthused with the broad antibody generation and robust T cell response generated by inactivated or live-attenuated virus, we propose a vaccine design comprising a cell membrane-coated nanoparticle, where the cell membrane presents the antigen and the nanoparticle carriers the adjuvant. In this study, we used a genetically engineered cell membrane expressing SARS-CoV-2 RBD to coat biodegradable mesoporous silica nanoparticles (MSNs) that are encapsulated with cytosine-phosphate-guanine oligodeoxynucleotide (CpG) (Supplementary Fig. S1). Such RBD-displaying nanovaccine mimics the multivalent surface display of specific antigens by a viral article, with an effective antigen presentation process potentiated by the encapsulated adjuvant. After establishing that this nanovaccine can be produced in scalable manner using a flash nanocomplexation (FNC) technique, we show that mice immunized with this nanovaccine developed high titers of SARS-CoV-2-neutralizing antibodies, along with robust protective immune responses.

We genetically fused the RBD to glycosylphosphatidylinositol (GPI)-anchor protein and used the transfected HEK 293 T cell to produce RBD-overexpressed membrane vesicle (Supplementary Fig. S2). In parallel, fluorescent-labeling GFP-RBD fusion membrane vesicles were prepared with more than 40% transfection efficiency (Supplementary Fig. S3). After encapsulating CpG into diselenide-bridged MSNs, we coated the CpG-loaded MSNs with RBD-displaying vesicles through either FNC or bulk sonication.^4^ Both core-shell structured nanovaccines (MSN-CpG@CM) exhibited uniform spike-like morphology in transmission electron microscopy (TEM) images with a size around 70 nm (Fig. 1a), which was close to that of SARS-CoV-2 virus particle (60–140 nm). The existence of RBD protein on the cell membrane of nanovaccine was determined by flow cytometry (Supplementary Fig. S3). The change of hydrodynamic size and Zeta potential compared with the bare MSN-CpG (Supplementary Fig. S4), along with the protein profile after cell membrane coating together demonstrated the display of RBD on the surface of these biomimetic nanovaccines (Fig. 1b). After determining their high antigen loading content (0.5 μg RBD per 100 μg nanovaccine), we showed that the biomimetic nanovaccines possessed long-term colloidal stability in phosphate buffer saline (PBS), as well as ROS-responsive degradation due to the presence of diselenide bond in the matrix of MSNs (Supplementary Fig. S5).^5^ We estimated that the nanovaccines can be produced at a rate of 2.4 g/h by using a MIVM with a total flow rate of 100 mL min^−1^.

**Fig. 1:**
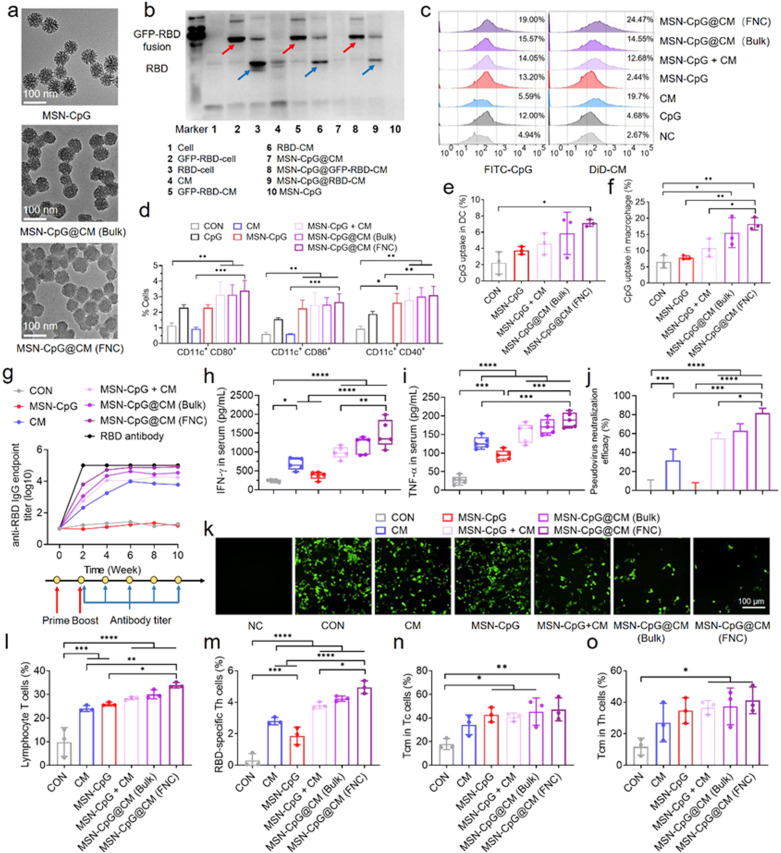
Scalable engineering of biomimetic SARS-CoV‑2 nanovaccines with robust protective immune responses. **a** TEM images of biomimetic nanovaccines. **b** Western blot analysis of RBD and GFP-RBD fusion expression on the cell membrane, cell membrane vesicles and nanovaccines. **c** Flow cytometry analysis of bone marrow-derived dendritic cells (BMDCs) internalization after 4 h of incubation with DiD-labeled cell membrane coated, FITC-labeled CpG loaded nanoparticles. **d** Quantification of DC maturation markers CD40, CD80 and CD86 after 24 h of incubation with nanovaccines through flow cytometry analysis. **e**, **f** Uptake of FITC-labeled nanovaccines by DCs (**e**) and macrophages (**f**) in the lymph node at 24 h after s. c. injection (*n* = 3). All the data were presented as mean ± SEM. **g** Total RBD-specific IgG antibodies overtime for nanovaccine-treated mice (*n* = 5). **h**, **i** Serum levels of IFN-γ (**h**) and TNF-α (**i**) were detected by ELISA (*n* = 5). **j** Neutralization activity of nanovaccine-immunized serum (*n* = 3). **k**. Fluorescence images of SARS-CoV-2 pseudovirus-infected HEK 293 T/ACE-2 cells after treatment with four-week immunized serum. (Scale bars, 100 μm). **l**, **m** Flow cytometry analysis of T lymphocytes (CD3 + ) (**l**) and RBD-specific Th cells (**m**) of four-week immunized mice. **n**, **o** Central memory T cells (CD44 + CD62L + ) of vaccinated mice was quantified as a percent of Tc (**n**) and Th (**o**) cells (*n* = 3). All data represent mean ± SEM; comparisons were made using one-way ANOVA with Tukey’s post-test

After confirming the biocompatibility of MSN-CpG@CM in two types of antigen-presenting cells (APCs) (Supplementary Fig. S6), we demonstrated that the FNC-produced nanovaccines had a higher uptake by bone-marrow-derived dendritic cells (BMDCs) than that of bulk-produced nanovaccines or membrane/adjuvant mixture (Fig. 1c and Supplementary Fig. S7). Both MSN-CpG@CM exhibited stronger DC maturation than that of the CpG or MSN-CpG group, coinciding with the greater secretion of TNF-α (Fig. 1d and Supplementary Fig. S8). Analysis of the lymph nodes revealed that the accumulation of nanovaccines in CD11c + DCs and F4/80+ macrophages was higher in MSN-CpG@CM group than the other groups (Fig. 1e, f and Supplementary Fig. S9), indicating that the nanovaccines could migrate to lymph nodes and be efficiently captured by APCs.

Encouraged by these findings, we immunized mice via *s.c*. injection and detected antibody titers every 2 weeks. All vaccination groups had strong RBD-specific antibody level within two weeks after the first dose, with the FNC-produced nanovaccines stimulating the highest responses (Fig. 1g and Supplementary Fig. S10). The titer of RBD-specific IgG for all vaccination groups increased until two weeks and maintained constant at a high level (10^5^) over the subsequent eight weeks. Notably, the RBD-specific IgG antibody titer level of mice vaccinated with only cell membrane vesicles was at 10^4^, suggesting the immunization potential of RBD-anchoring cell membrane. The strong humoral immune response of the FNC-produced MSN-CpG@CM group was reflected in elevated serum levels of TNF-α and IFN-γ in the 4-week-immunized mice (Fig. 1h, i). Next, we assessed the neutralizing capability of the immunized sera by determining the efficiency against SARS-CoV-2 pseudovirus infection of HEK 293 T cells, which over-expressed the angiotensin-converting enzyme 2 (ACE2). As shown in Fig. 1j, k, serum samples from the FNC-produced nanovaccine group exhibited more than 80% neutralization efficacy, as characterized by a significant reduction in viral particles. In contrast, the serum from the bulk-produced nanovaccine and cell membrane vesicle groups presented a weaker blocking effect of about 70 and 30%, respectively. These findings suggested that the biomimetic nanovaccine might induce an enhanced neutralizing antibody response against SARS-CoV-2.

We next proceeded to evaluate the enhancement of the T cell immunity by investigating the isolated cells from the spleens of 4 week-immunized mice. Compared to the control group, splenocytes from all the immunized mice showed a high proportion of T lymphocytes (CD3 + ), ranging from 25 to 35% (Fig. 1l). The FNC-produced nanovaccines presented the highest ratio of RBD-specific Th cells. In contrast, other nanovaccines exhibited less proportion of RBD-specific Th cells (Fig. 1m), demonstrating the RBD-specific response of biomimetic nanovaccine-based immunization. Both cytotoxic T cells (CD3 + CD8a + CD4-) and helper T cells (CD3 + CD4 + CD8a-) in the spleen of vaccinated mice exhibited an increase of 7%-20%, with a high proportion (50%) of them were the central memory T cells (CD44 + CD62L + ), which would account for a quick immune response when exposed to the same antigen again (Fig. 1n, o and Supplementary Fig. S11–13). Finally, no major alterations in body weight or blood biochemical parameters were observed at the end of the vaccination (Supplementary Fig. S14). Hematoxylin and eosin (H&E) staining images further confirmed no noticeable histopathological changes in the major organs (Supplementary Fig. S15), suggesting an acceptable safety profile of the nanovaccines.

In summary, we have engineered biomimetic nanovaccines with robust protective immune responses in a facile and manufacturable manner. RBD-displaying nanovaccines elicited a potent humoral and cellular immune response in mice with less safety concerns. This study suggests that a vaccine candidate comprising a genetically engineered cell membrane coating a nanoparticle core loaded with a variety of adjuvant can be rapidly constructed against any viral infection, an alternative vaccine design that might be valuable for a pandemic like the one we have been facing. Importantly, there was only approximately 0.5 μg RBD protein delivered to each mouse in our current study, which was less than what other studies used to achieve similar RBD-specific antibody titers (Supplementary Table. S1). We are investigating if this low-cost and versatile vaccine design would be effective on the mouse model of SARS-CoV-2 infection.

## Supplementary information

Supporting Information

## References

[1] Chen, W. H., Strych, U., Hotez, P. J. & Bottazzi, M. E. The SARS-CoV-2 Vaccine Pipeline: an Overview. *Curr. Trop. Med. Rep.* **3**, 1–4 (2020). 10.1007/s40475-020-00201-6 PMC7094941 32219057

[2] Pierri CL (2020). SARS-CoV-2 spike protein: flexibility as a new target for fighting infection. Signal Transduct. Target Ther..

[3] Wu, Y. et al. A recombinant spike protein subunit vaccine confers protective immunity against SARS-CoV-2 infection and transmission in hamsters. *Sci. Transl. Med.* **13**, eabg1143 (2021). 10.1126/scitranslmed.abg1143 PMC9836081 34285130

[4] Hu H (2021). A Versatile and Robust Platform for the Scalable Manufacture of Biomimetic Nanovaccines. Adv. Sci. (Weinh.).

[5] Shao D (2020). Biomimetic Diselenide-Bridged Mesoporous Organosilica Nanoparticles as an X-ray-Responsive Biodegradable Carrier for Chemo-Immunotherapy. Adv. Mater..

