# Supplementary material for: Scalable biomimetic SARS-CoV‑2 nanovaccines with robust protective immune responses
Source: Signal Transduct Target Ther. 2022 Mar 25;7:96. doi: 10.1038/s41392-022-00942-y (PMC8949828; doi:10.1038/s41392-022-00942-y)
Supplement: Supplementary file 1 — Supporting Information [file 41392_2022_942_MOESM1_ESM.pdf]

Supplementary Materials for

Scalable Biomimetic SARS-CoV-2 Nanovaccines with Robust Protective Immune Responses

Xuenian Chen[#1,3,4], Tongfei Shi[#1,4], Chao Yang*[1,2,4], Fangman Chen[1], Xuan He[1,4], Kunbao Zhang[1,4], Hanze Hu[2], Lulu Cai[5], Kam W. Leong*[2] and Dan Shao*[1,3,4,6,7]

[1] School of Biomedical Sciences and Engineering, South China University of Technology, Guangzhou International Campus, Guangzhou, Guangdong 511442, China
[2] Department of Biomedical Engineering, Columbia University, New York, NY 10027, USA
[3] Institutes of Life Sciences, School of Medicine, South China University of Technology, Guangzhou, Guangdong 510006, China
[4] National Engineering Research Center for Tissue Restoration and Reconstruction, South China University of Technology, Guangzhou, Guangdong 510006, China
[5] Personalized Drug Therapy Key Laboratory of Sichuan Province, Department of Pharmacy, Sichuan Provincial People's Hospital, School of Medicine, University of Electronic Science and Technology of China, No. 4, Section 2, Jianshe North Road, Chengdu, 610072, China
[6] Guangdong Provincial Key Laboratory of Biomedical Engineering, South China University of Technology, Guangzhou 510006, China
[7] Key Laboratory of Biomedical Material and Engineering of the Ministry of Education, South China University of Technology, Guangzhou 510006, China

# These authors contributed equally.
* Correspondence: Chao Yang, Email: chaoyang@scut.edu.cn,
Kam W. Leong, Email: kamleong@columbia.edu,
Dan Shao, Email: shaodan@scut.edu.cn

This PDF file includes:

Materials and Methods
Figures. S1 to S15
Tables S1 to S3
References

Content

I. Materials and methods 3
1. Materials 3
2. Construction and transfection of the plasmids 3
3. Synthesis of biomimetic coronavirus nanovaccine 4
4. Characterization of biomimetic coronavirus nanovaccine 5
5. Cell culture 5
6. Cytotoxicity assay 6
7. In vitro uptake and activity 6
8. Animal 7
9. In vivo lymph node distribution 8
10. Mouse vaccination experiments 8
11. RBD-specific IgG antibody detection and neutralization test of pseudovirus 9
12. Statistical analysis 10
II. Extended figures and tables 11
Figure S1. Schematic illustration 11
Figure S2. Construction and qualification of transfected plasmids. 12
Figure S3. Plasmid transfection efficiency of HEK293T cells and cell membrane coating efficiency. 13
Figure S4. Hydrodynamic characterization and stability of nanovaccines. 14
Figure S5. Stability, degradability and antigen quantification of nanovaccines 15
Figure S6. Biocompatibility of nanovaccines. 16
Figure S7. Quantification of CD40, CD80 and CD86 expression 17
Figure S8. Uptake of nanovaccines in APCs. 18
Figure S9. APCs in the inguinal lymph node at 24 h after foot injection of nanovaccines. 19
Figure S10. ELISA absorbance vs. dilution curves. 20
Figure S11. Enhancement of the T-cell response by nanovaccines in vivo 21
Figure S12. Representative T lymphocytes and APCs gating strategy. 22
Figure S13. Flow cytometry analysis of portion of APCs, T lymphocytes and their subtypes 23
Figure S14. Biological safety of nanovaccines in vivo 24
Figure S15. Histological safety of nanovaccines. 25
Table S1. Comparation of our work with others typical reports 26
Table S2. Antibodies used for Flow Cytometer experiments of immune cells in the spleen of vaccinated mice. 27
Table S3. Antibodies used for Flow Cytometry experiments of uptake by APCs in the lymph nodes. 28
III. References 29

I. Materials and methods

1. Materials

Tetraethyl orthosilicate (TEOS), bis[3-(triethoxysilyl)propyl]tetrasulfide (BTESPT), γ-chloropropyl trimethoxysilane (CP), 3-aminopropyltriethoxysilane (APTES), etyltrimethylammonium tosylate (CTAT), triethanolamine (TEAH3), triethanolamine (TEA), carboxyl-terminated 50:50 poly(lactic-co-glycolic) acid, succinic anhydride and silica dioxide microparticles with micron were purchased from Sigma-Aldrich Co. (St Louis, MO, USA). Vybrant DiD Cell-Labeling Solution (V22887) was purchased from Thermo Fisher Scientific. FITC labeled-CpG ODN 1826 was purchased from Sangon Biotech. Diselenide bridged MSN with applicable pore size, and surface charge were synthesized according to our previous reports [5]. TNF-α, IFN-γ (mouse) ELISA Kit were bought from Elabscience (Wuhan, China). Antibodies used in the experiment of flow cytometry were obtained from Biolegend (California) or BD (USA) as shown in Table 2 and Table 3. SARS-CoV-2 Spike pseudovirus were bought from OBiO Technology (Shanghai) Corp. Ltd. SARS-CoV-2 Spike RBD Antibody in Western Blot was bought from R&D Systems (USA). Secondary antibody PE/Dazzle™ 594 Goat anti-mouse IgG Antibody for RBD quantification was bought from Biolegend (California). HEK293T-ACE2 cells, IL-4, and GM-CSF, were bought from Novoprotein (China).

2. Construction and transfection of the plasmids

The RBD expression sequence fragment was GPI anchor-linker-RBD, and the GFP-RBD expression sequence fragment was GPI anchor-linker-GFP-linker-RBD. The

RBD is a gene encoded the residues 319 – 541 aa of SARS-CoV-2 (YP_009724390.1), and the GFP (GenBank: ABG78037.1) is 239 aa length, GPI length was 44 aa derived from GPIHBP1 (GenBank: AAH63857.1). Linker was chosen as Gly-Gly dipeptide. The two fragments were cloned into the pcDNA3.4 vector. HEK293T cells were transfected with plasmids independently by lipofectamine 3000 reagent.

3. Synthesis of biomimetic coronavirus nanovaccine

The CpG loading was achieved with the MSN-to-CpG mass ratio of 5 to 1. The MSN-CpG was obtained after stirring at 4 °C overnight. Cell membrane extraction experiment was conducted as follows, after trypsinization, HEK293T cells were lysed and homogenized by sonication, then the cell pellets were collected by ultracentrifugation, finally suspended in DI water. The total membrane protein contents were quantified using the BCA protein assay kits.

FNC and bulk sonication producing-nanoparticles were synthesized as follows: all particles and cell membrane fragments were well dispersed in DI water, respectively. In the FNC method, particle solutions and cell membrane fragments were introduced into different inlets of the MIVM, respectively. The flow rate of 30 mL/min was applied to prepare membrane-coated particles with a mass ratio of 1/1. The efflux was collected for further use. For nanoparticles coated using the bulk sonication method, equal volumes of cell membrane vesicles and particle cores were mixed, pipetted, and sonicated. Nanoparticles of mixture formulation were prepared by mixing the CpG loaded MSN and cell membrane at the equal mass after dispersion.

4. Characterization of biomimetic coronavirus nanovaccine

The size, polydispersity index (PDI) and zeta potential of naked MSN, cell membrane, CpG loaded MSN, cell membrane-coated nanoparticles of FNC, bulk sonication and mixture formulation were measured using a Malvern Zetasizer. To assess the stability of CpG coated MSN, membrane-coated nanoparticles of FNC and bulk sonication formulation, particles were stored in the FBS containing DMEM medium and PBS for 8 days and measured by DLS every day. For TEM characterization, samples were prepared and dried onto a carbon-coated copper grid. Identification of RBD protein expressed on the cell membrane or coated on the NPs was performed by Western blotting. To quantify the expression of RBD protein coated on the NPs, a range of concentrations of RBD protein and NPs was performed by Western Blot. To quantify the efficiency of plasmid transfection and NPs coating, two parallel fluorescence methods of RBD antibody-conjugated PE and GFP-RBD fusion protein were quantified by flow cytometry.

5. Cell culture

Human embryonic kidney cells, HEK 293T (CRL-3216), were cultured for cell membrane derivation. Cells were cultured in DMEM media with 10% fetal bovine serum (Gibco) and 100 U penicillin-streptomycin. The generation of bone-derived Dendritic cells (BMDCs) followed a previously published protocol. Healthy mice were euthanized using carbon dioxide asphyxiation followed by cervical dislocation. Both

femurs and tibias were dissected, cleaned in 75% ethanol, and cut on both ends. Bone marrow was then flushed out of the bone with a 1 mL sterile syringe using warm PBS. Cells were then pelleted at 700×g for 5 min, resuspended in a certain amount of red blood cell lysis buffer to reduce the red blood cell. Cells were then pelleted at 700×g for 5 min again, resuspended in BMDC growth media, consisting of the basal media further supplemented with 20 ng/mL granulocyte/macrophage-colony stimulating factor (GM-CSF) and 10 ng/mL interleukin-4 (IL-4), to a concentration of 1×10^6 cells/mL, and plated into Petri plates at 10×10^6 cells per 100 mm plate. The medium was half-changed every two days.

6. Cytotoxicity assay

The cytotoxicity of naked MSN, cell membrane, CpG coated MSN, cell membrane-coated nanoparticles of bulk sonication, FNC and mixture formulation in the RAW264.7 cells or BMDCs was assessed using a CCK8 assay. The cells in the proper density were cultured in complete medium containing 5 or 20 μg/mL substances (CpG formulation) for 24 h, and then incubated with CCK8 solution for 1 hour. The cell activity was detected by reading the OD at 450 nm.

7. In vitro uptake and activity

For the cellular uptake study, BMDCs were collected on day 5 and then plated into 12-well plates. FITC-labeled CpG, DiD-labeled CM, MSN-CpG, CM-coated nanoparticles of FNC, bulk and mixture formulation were added at an equivalent CpG concentration

of 5 μg/mL. After 4 h incubation, the cells were washed and stained with DAPI. 15 min later, cells were imaged by fluorescence. For flow cytometry, cells were collected, washed twice in PBS, and resuspended in 300 μL PBS. The cell suspension was analyzed using BD FACSCelesta flow cytometer. The activity of the delivered CpG was examined using a BMDC maturation assay and cytokine release assay. BMDCs were collected on day 5, and 5×10^5 BMDCs were plated into 6-well plates in BMDC growth media. Cells were pulsed with materials for 12 h at 5 μg/mL CpG, then washed twice with fresh media. After an additional 24 h of culture, cell supernatants were collected and cytokine content was analyzed using TNF-α ELISA kits. The cells were then collected, washed twice and stained with PE-conjugated CD11c, PerCP/Cy5.5-conjugated CD40, PE/Dazzele 594-conjugated CD80 and APC-conjugated CD86. Data were collected using a BD FACSCelesta flow cytometer. RAW264.7 cells were plated into 96-well suspension plates at 2×10^4 cells/well and pulsed with materials for 24 h at 5 μg/mL CpG, then cell supernatants were collected and cytokine content was analyzed using TNF-α ELISA kits.

8. Animal

All animals received care in compliance with the guidelines outlined in the Guide for the Care and Use of Laboratory Animals, and the procedures were approved by the South China University of Technology Animal Care and Use Committee. Female BALB/c mice were obtained at 5 weeks old from Hunan SJA Laboratory Animal Co., LTD.

9. In vivo lymph node distribution

FITC-labeled MSN-CpG, mixture, CM-coated nanoparticles of FNC and bulk formulation were used for the antigen persistence at lymph nodes. At 12 h after injecting 20 μL of different NPs at foot pad, mice were euthanized and their inguinal lymph nodes were collected, stained with antibodies for dendritic cells and macrophages (panel shown in Table S2) for 30 min. Data were collected using BD FACSCelesta flow cytometer and analyzed using FlowJo software.

10. Mouse vaccination experiments

Female 5-week-old BALB/c mice were randomly assigned to 5 cohorts of MSN-CpG, cell membrane, mixture, CM-coated nanoparticles of FNC and bulk formulation, vaccinated with about 500 ng of RBD subcutaneously and boosted on week 2, respectively. Mice of control group were injected with normal saline of equal volume. The weight of mice were monitored every week. On week 2, 4, 6, 8, 10 after prime vaccination, mice were sacrificed and blood was collected in the coagulation-promoting tubes. Plasma was separated by centrifugation and stored at -80 °C for further use. The levels of TNF-α and IFN-γ in serum of vaccinated mice on week 4 were detected by ELISA kits.

To assess RBD-specific T cells, mice were sacrificed on the week 4 and splenocytes were collected. Single-cell suspensions of splenocytes were prepared by gently grinding. Splenocytes were stained for lymphocyte, macrophages, DCs, Tc cells,

Th cells and the subtypes (panel shown as Table S1) for 30 min. Then, stained cells were incubated with 10 μg/mL DiD-labeled MSN-CpG@CM (FNC) binding to the RBD-specific T cells. Data were collected using BD FACSCelesta flow cytometer and analyzed using FlowJo software.

To evaluate the safety of vaccines, the main tissues including livers, kidneys, spleens, lungs and hearts from the vaccinated mice on week 10 were collected, fixed in 4% formalin and sectioned for hematoxylin and eosin (H&E) staining. The biochemical parameters including alanine aminotransferase (ALT), aspartate aminotransferase (AST), Urea, creatinine (CREA) and total protein (TP) were assayed.

11. RBD-specific IgG antibody detection and neutralization test of pseudovirus

Antibody titer detection adopted indirect ELISA method. In short, RBD protein (1 μg/ml) was coated on the well plate and stored overnight at 4 ℃. And then plate was washed three times. The serum of vaccinated mice on week 2, 4, 6, 8, 10 were serial diluted and added into the pre-coated well plate for incubation (37 ℃, 1 h). And then the PBS was added to wash the well three times. Next, the enzyme labeled antibody was added and incubated for one hour. And then the PBS was added to wash the well three times. TMB solution was added to incubate for near 15 minutes in the dark. Finally, the stop solution was added, and the absorbance at 450 nm was measured with a microplate reader. The absorbance data was simulated in nonlinear fitting by Origin 2021b software. The positive data was confirmed 2.1 times larger than the negative data.

In the pseudovirus neutralization test, 20 μL of serum of vaccinated mice on week 4 was first added and incubated with 20 μL of SARS-CoV-2 Spike pseudovirus with GFP expressing the gene for 30 min and then the mixture was added to HEK293T-ACE2 cells to 200 μL total culture medium. After 24 h of co-cultivation, the intracellular fluorescence content was measured by fluorescence to verify the protective effect of the serum RBD-specific antibody against pseudovirus.

12. Statistical analysis

Statistical analysis was performed using GraphPad Prism 9 (GraphPad). Data were analyzed using one-way ANOVA with Tukey's post-hoc correction for multiple hypothesis testing unless otherwise stated. All flow cytometry data were analyzed using FlowJo_v10.7.2 software (FlowJo LLC, BD Biosciences).

II. Extended figures and tables

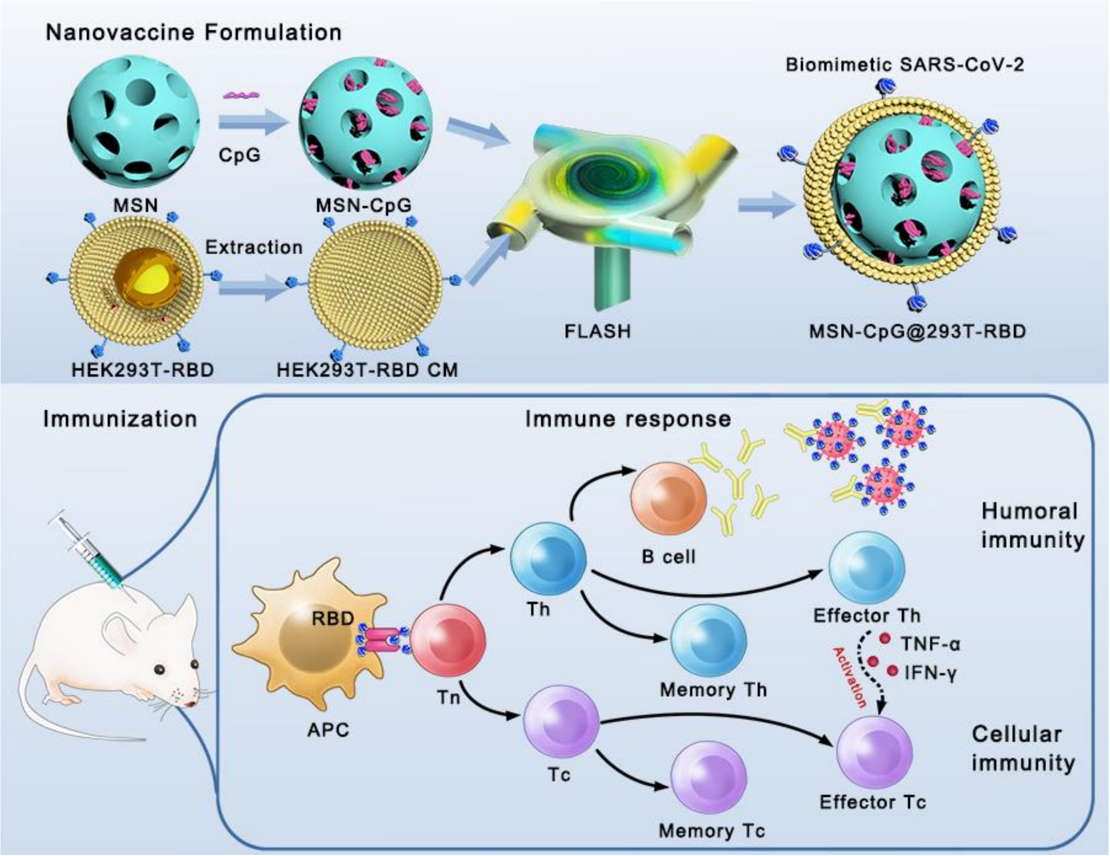

Figure S1. Schematic illustration of the synthesis procedure for the scalable engineered biomimetic SARS-CoV-2 nanovaccines and their effects on boosting humoral immunity and cellular immunity.

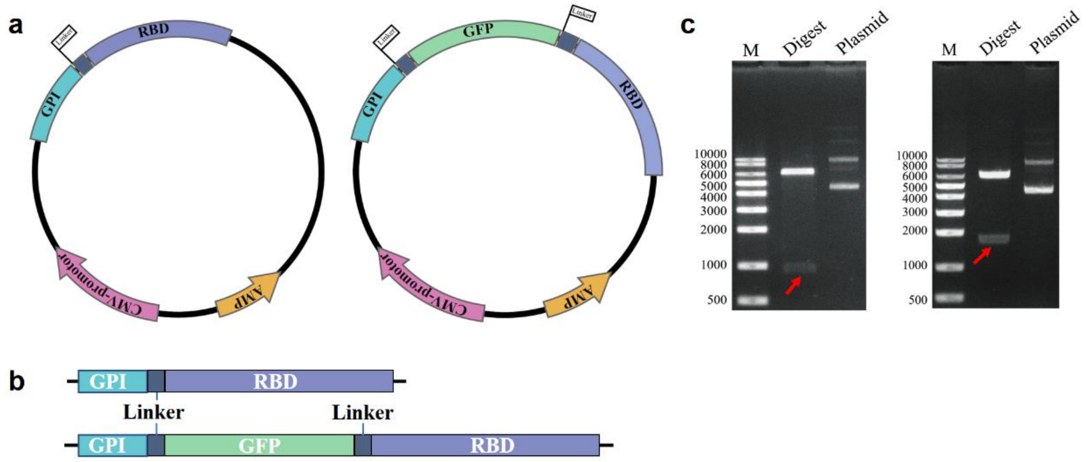

Figure S2. Construction and qualification of the transfected plasmids. a) Schematic illustration of two constructed plasmids based on the pcDNA3.4 frame. pcDNA3.4-RBD (left) and pcDNA3.4-GFP-RBD (right). b) The construction of two gene fragments, the RBD fragment (above) and the GFP-RBD fragment (below). c) Electrophoresis of two plasmids with double enzyme digestion, pcDNA3.4-RBD (left) and pcDNA3.4-GFP-RBD (right).

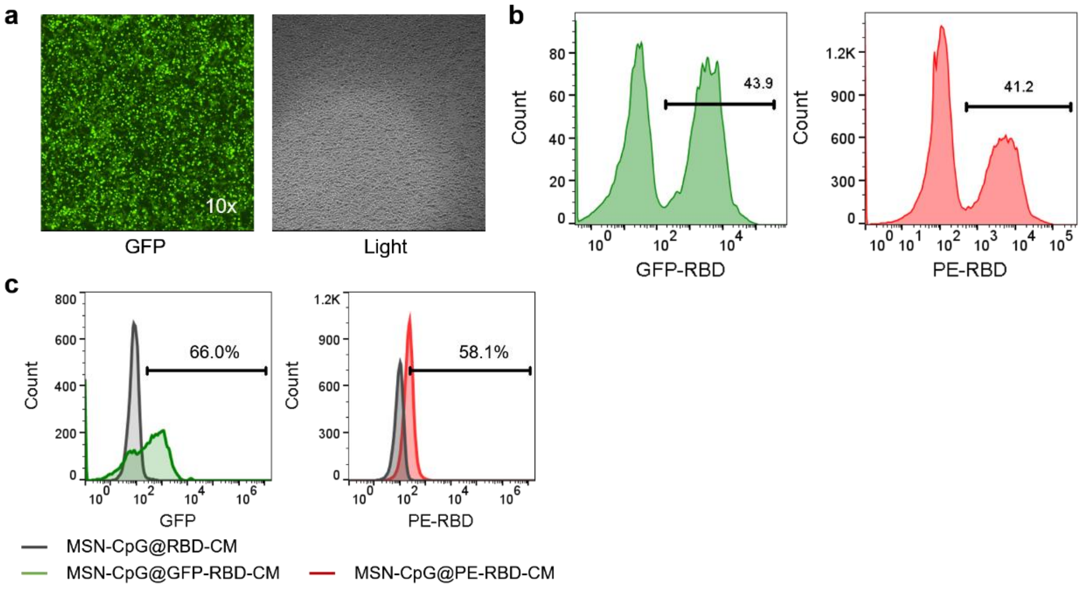

Figure S3. Plasmid transfection efficiency of HEK293T cells and cell membrane coating efficiency. a) Fluorescence image of HEK293T cells transfected with GFP-RBD fusion plasmid. b) Quantification of plasmid transfection efficiency via GFP-RBD fusion (left) or PE-labeled RBD protein (right) on the cell surface by Flow cytometry. c) Quantification of coating efficiency in FNC-produced nanoparticles via

GFP-RBD fusion (left) or PE-labeled RBD protein (right) on the cell membrane by Flow cytometry.

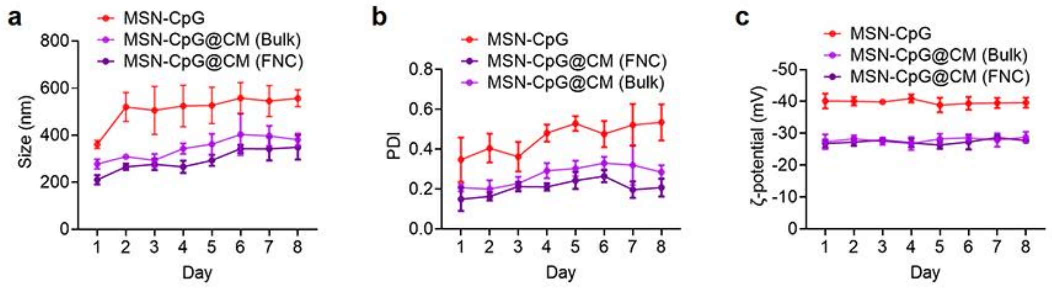

Figure S4. Hydrodynamic characterization and stability of nanovaccines. Time-dependent a) Size, b) PDI and c) Zeta-potential of bulk sonication and FNC formulation compared to MSN-CpG nanoparticles in PBS solution during 8 days. Data represent mean ± SEM (n=3).

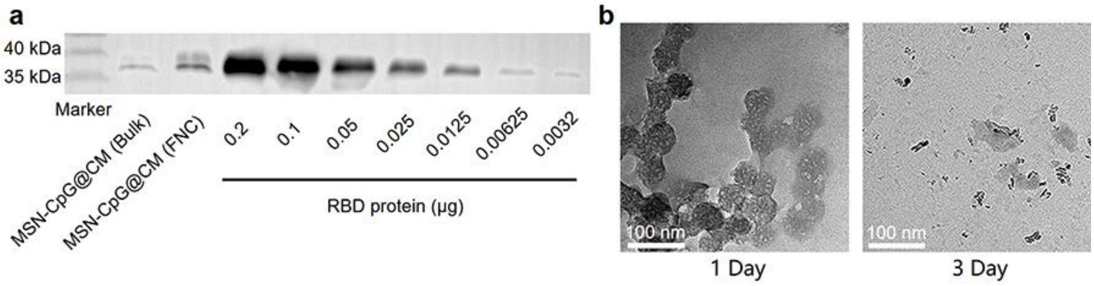

Figure S5. Antigen quantification and degradability of nanovaccines. a) Quantification of RBD protein on the surface of nanoparticles from bulk sonication and FNC formulation. b) TEM images of FNC nanoparticles after 1-day and 3-day incubation in simulated body fluid solution containing 100 μM H_2O_2.

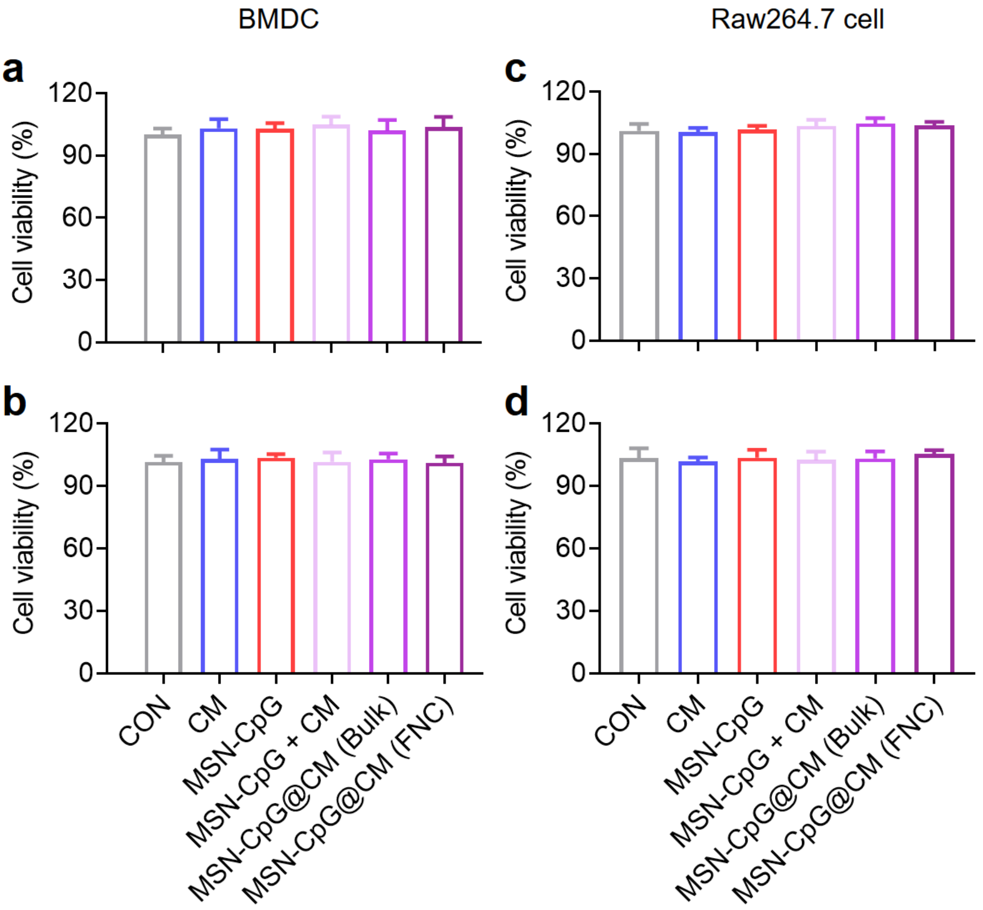

Figure S6. Biocompatibility of nanovaccines. The viability of a and b) BMDCs and c and d) RAW264.7 after treatment with different nanoparticles (5 μg/mL (above) or 20 μg/mL (below) CpG formulation) for 24 h. Data represent mean ± SEM (n=3).

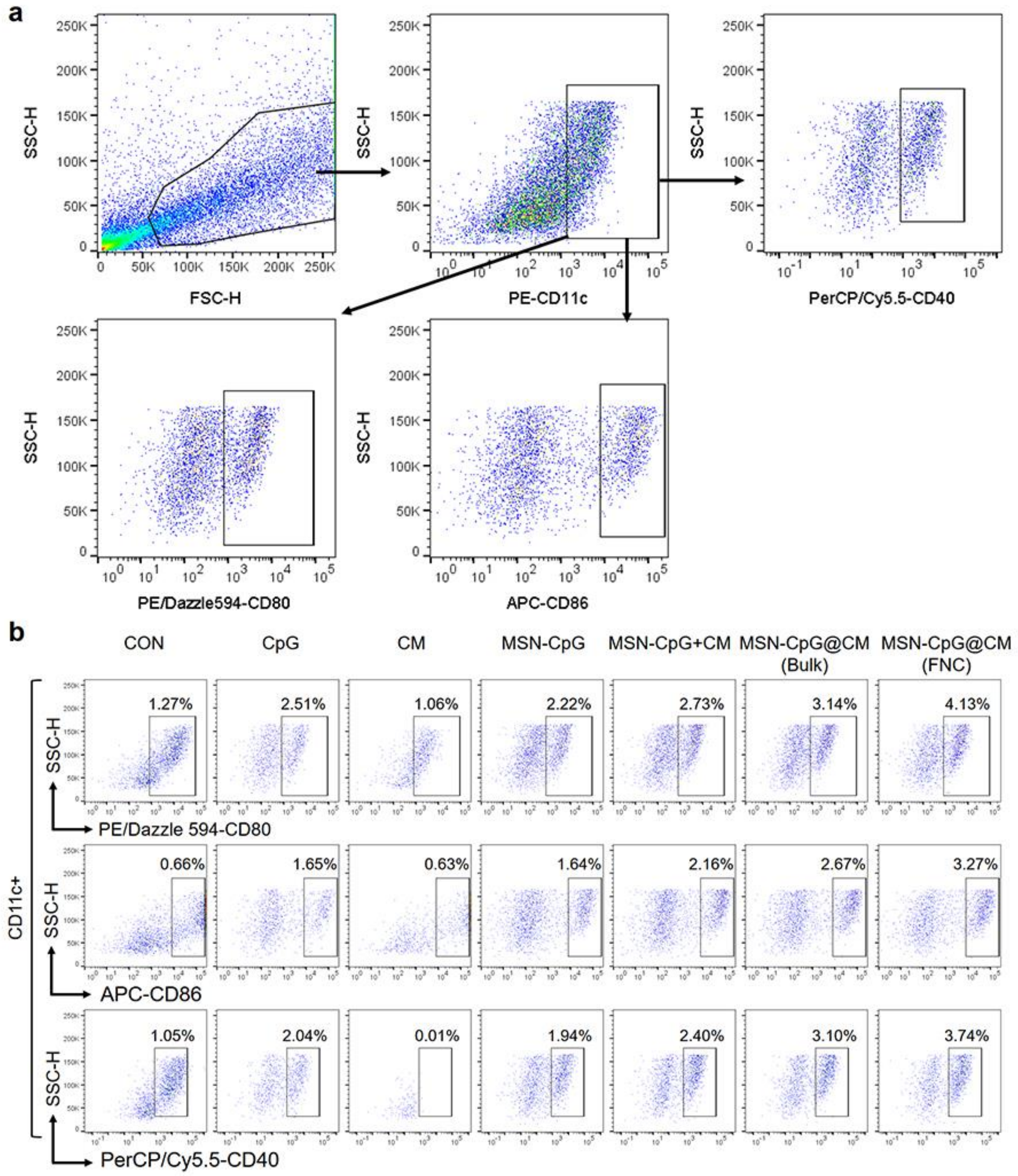

Figure S7. Quantification of CD40, CD80 and CD86 expression, *which are markers for DC maturation on the surface of DCs (CD11c+) after in vitro incubation with nanovaccines for 24 h by Flow cytometry. a) Representative BMDCs gating strategy. b) Flow cytometry analysis of portion of CD40, CD80 and CD86 expressed in BMDCs treated with different nanovaccines and materials in vitro.*

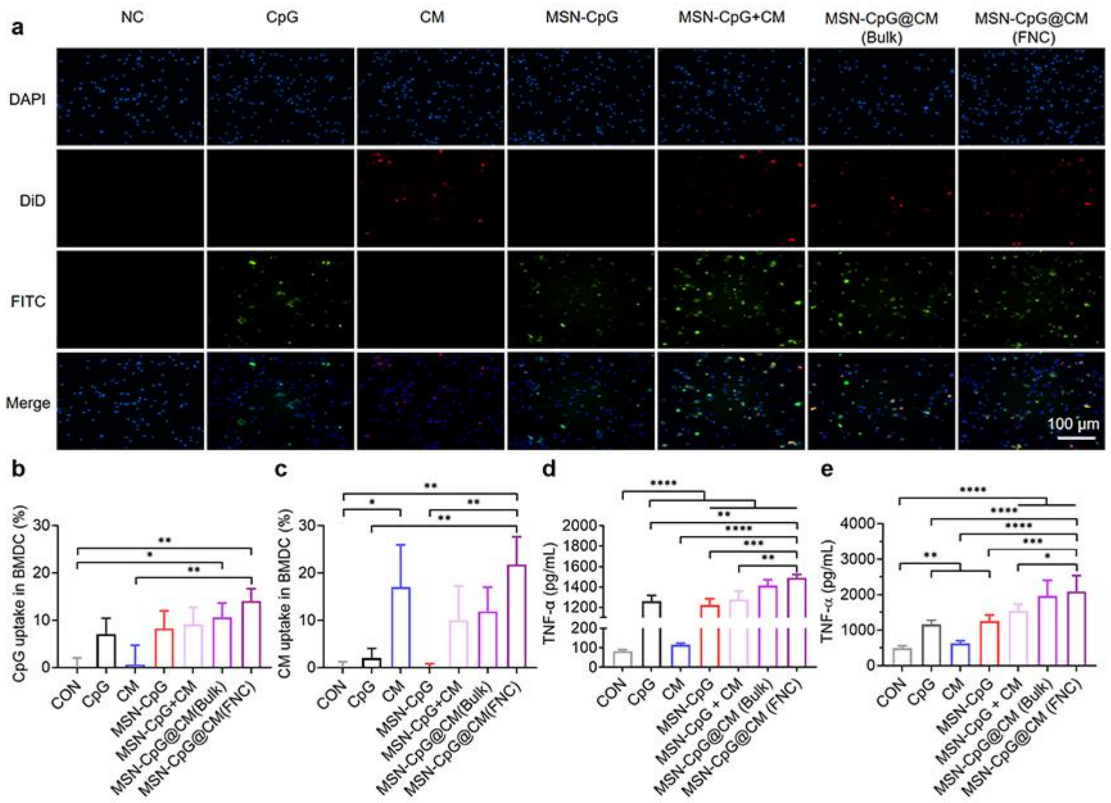

Figure S8. Uptake of nanovaccines in APCs. a) Intracellular colocalization of FITC-labeled CpG loaded and DiD-labeled cell membrane-coated nanoparticles by BMDCs. Quantification of fluorescence intensity of b) FITC or c) DiD in Figure 1c. TNF-α secretion of d) BMDC and e) RAW264.7 cells treated with nanovaccines (5 μg/mL CpG) were assayed via ELISA. Data are means ± SEM (n=3 independent experiments; *P<0.05, **P<0.01, ***P<0.001, ****P<0.0001 by one-way ANOVA with Tukey's multiple comparison test). Scale bar, 100 μm.

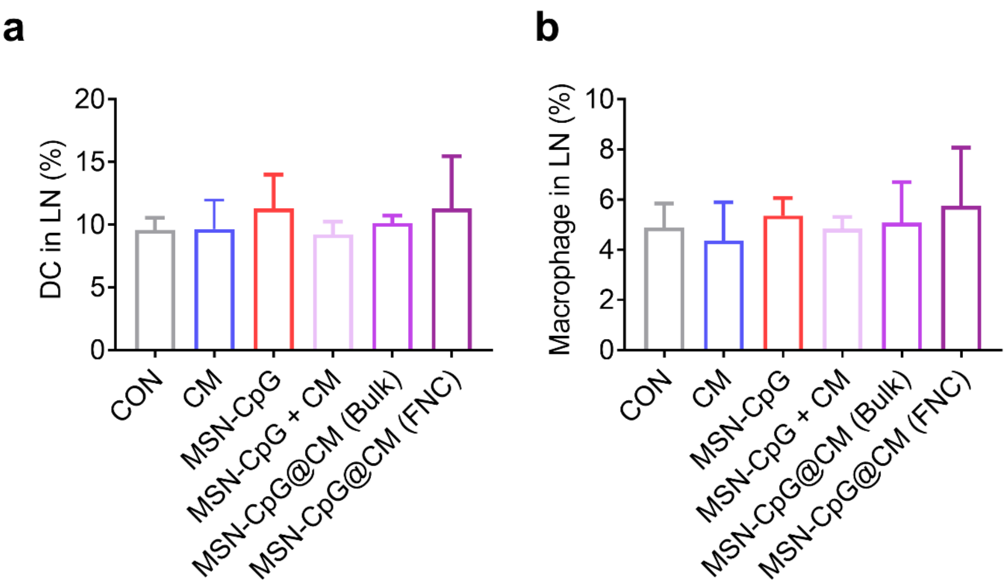

Figure S9. APCs in the inguinal lymph node at 24 h after foot injection of nanovaccines. a) DCs and b) macrophages quantified as a percentage of total cells in the inguinal lymph node. Data are means ± SEM (n=3).

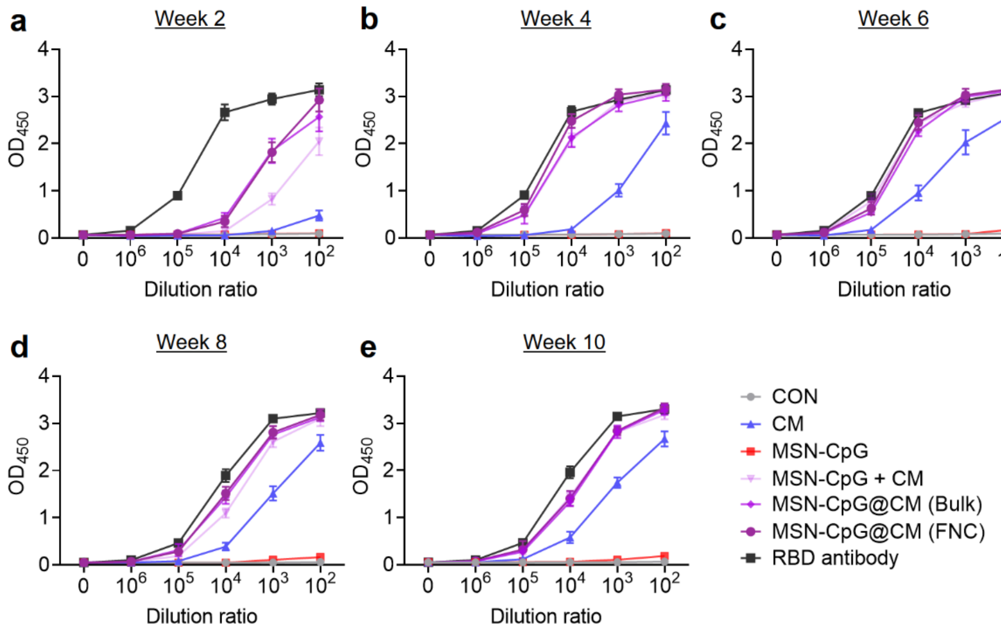

Figure S10. ELISA absorbance vs. dilution curves. Absorbance vs. dilution for RBD-specific ELISAs on a) week 2, b) week 4, c) week 6, d) week 8, e) week 10. Data are means ± SEM (n=5 mice per group).

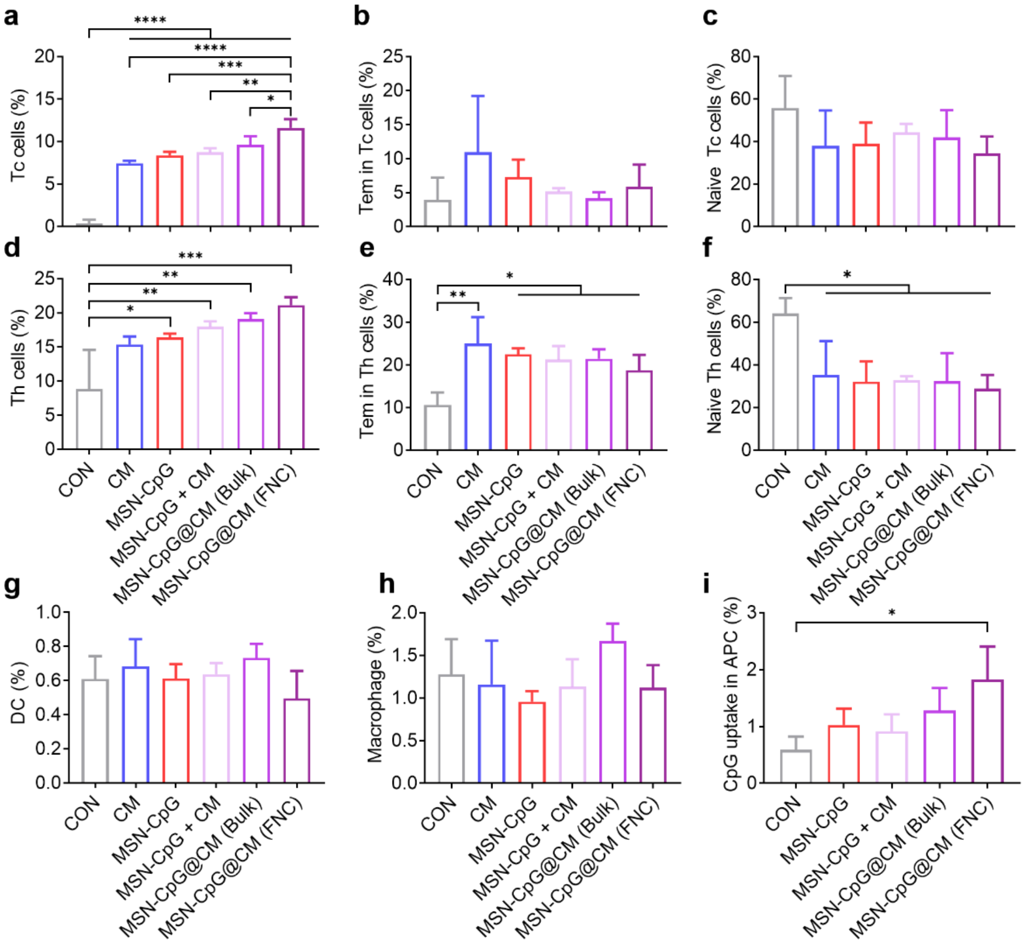

Figure S11. Enhancement of the T-cell response by nanovaccines in vivo. a) Cytotoxic T cells (CD3+ CD8a+ CD4-), d) CD4+ helper T cells (CD3+ CD4+ CD8a-) in the spleen of vaccinated mice of week 4. b) and e) Effective Memory T cells (CD44+ CD62L-), c) and f) Naïve T cells (CD44- CD62L+) of vaccinated mice quantified as a percent of Tc and Th cells, respectively. g) DCs and h) macrophages of immunized mice quantified as a percentage of spleen immune cells. Uptake of FITC-labeled CpG loaded nanoparticles of different formulations by i) APCs in the inguinal lymph node at 24 h after injection. Data are means ± SEM (n=3 independent experiments; *P<0.05, **P<0.01, ***P<0.001, ****P<0.0001 by one-way ANOVA with Tukey's multiple comparison test).

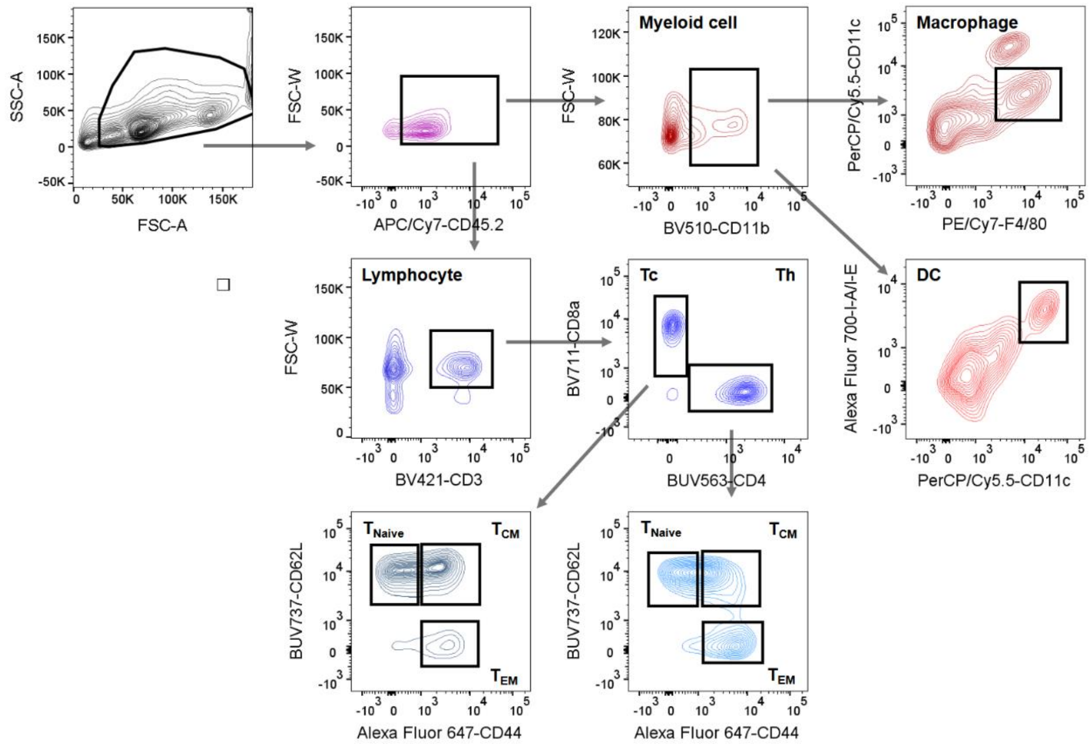

Figure S12. Representative T lymphocytes and APCs gating strategy.

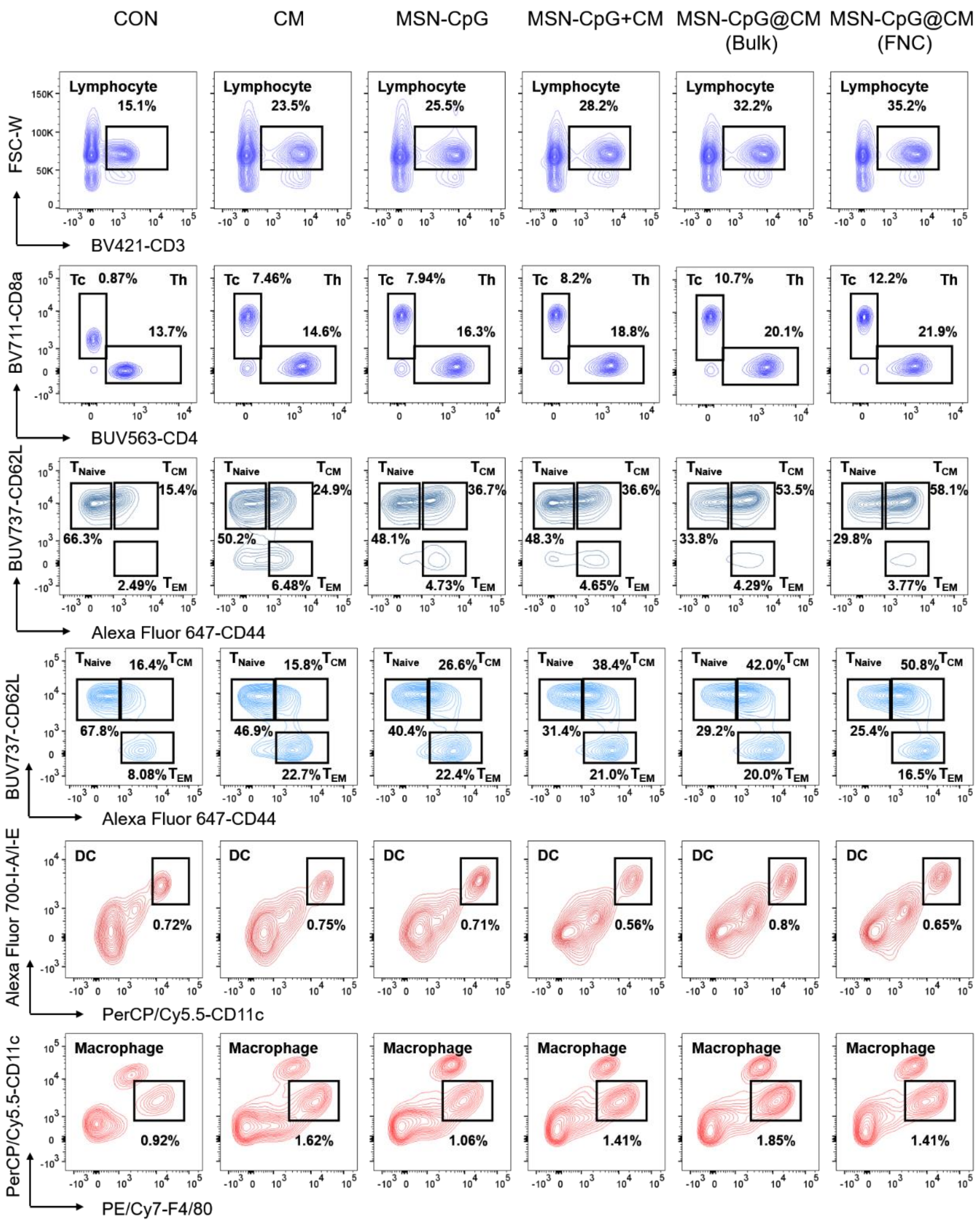

Figure S13. Flow cytometry analysis of portion of APCs, T lymphocytes and their subtypes of vaccinated mice treated with different nanovaccines **in vivo.**

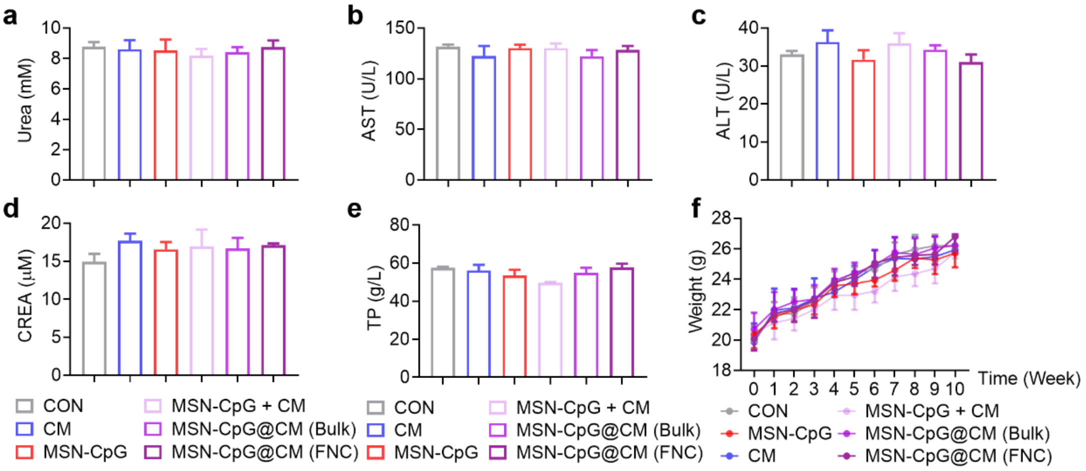

Figure S14. Biological safety of nanovaccines in vivo. a) Urea, b) AST, c) ALT, d) CREA, and e) TP were measured in serum on week 10. Data represent mean ± SEM (n=3). f) Body weight was monitored until 10 weeks after the prime vaccination. Data represent mean ± SEM (n=5).

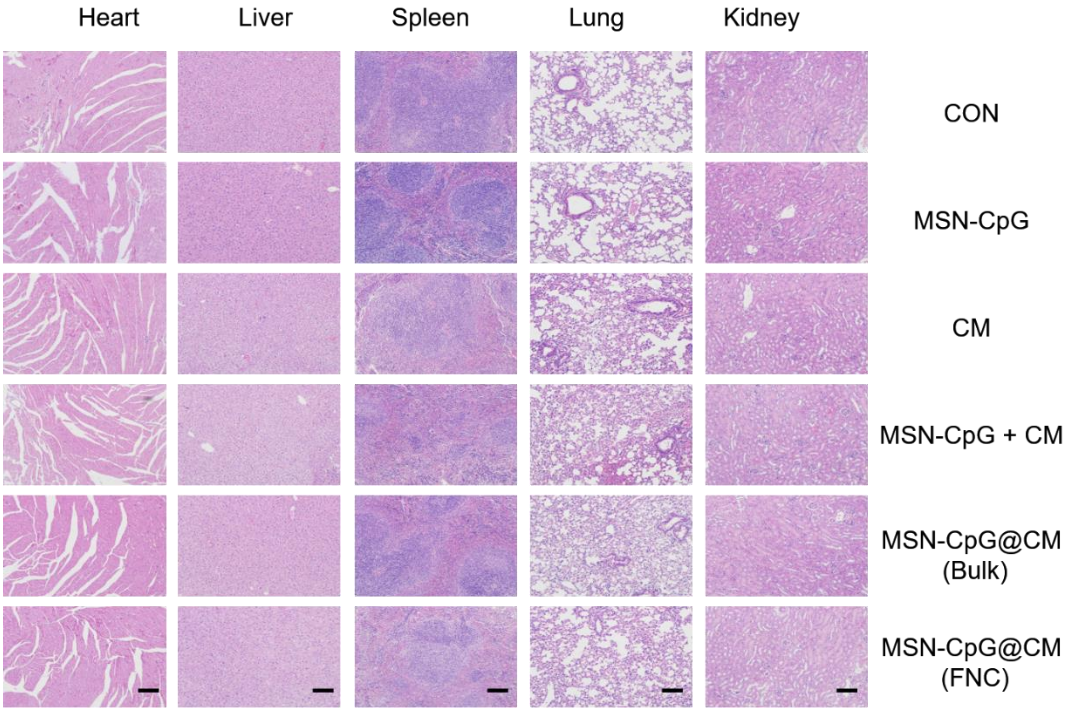

Figure S15. Histological safety of nanovaccines. Histological images from the major organ slices of mice administrated with nanoparticles of different formulations. Scale bar, 100 μm.

***Table S1. Comparation of our work with others typical reports*.**[1-5]

	**Ligation method**	**Antibody titer (log10)**	**Detection time**	**Antigen dosage/per mouse per injection**	**Injection times**	**References**
1	GPI anchor	4-5	2-10 weeks	0.5 μg RBD	2	Our Nanovaccine
2	Charge interaction	4-6	2-10 weeks	20 μg S1	3	1
3	Covalently conjugate	4-5	2-10 weeks	10 μg RBD	2	2
4	Affinity interaction	4	2-4 weeks	0.1 μg RBD	2	3
5	Electrostatic interaction	3-4	8 weeks	10 μg RBD	2, 3	4
6	Chemical coupling	3-4	4 weeks	10 μg RBD	2	5

Table S2. Antibodies used for Flow Cytometer experiments of immune cells in the spleen of vaccinated mice.

Specificity	Fluorochrome	Cat #	Source
Alexa Fluor 647	CD44	103018	Biolegend
APC/Cy7	CD45.2	109824	Biolegend
Alexa Fluor 700	I-A/I-E	107622	Biolegend
BV421	CD3	100228	Biolegend
BV711	CD8a	100748	Biolegend
PerCP/Cy5.5	CD11c	117328	Biolegend
V500	CD11b	562127	BD
BV563	CD4	612923	BD
BV737	CD62L	612833	BD
PE/Cy7	F4/80	123114	Biolegend

Table S3. Antibodies used for Flow Cytometry experiments of uptake by APCs in the lymph nodes.

Specificity	Fluorochrome	Cat #	Source
APC/Cy7	CD45.2	109824	Biolegend
PE	CD11c	117318	Biolegend
Alexa Fluor 700	I-A/I-E	107622	Biolegend
PerCP/Cy5.5	Ly-6c	128012	Biolegend

III. References

1 Liu, L. *et al.* Subunit Nanovaccine with Potent Cellular and Mucosal Immunity for COVID-19. *ACS Applied Bio Materials* **3**, 5633-5638 (2020).

2 Ma, X. *et al.* Nanoparticle Vaccines Based on the Receptor Binding Domain (RBD) and Heptad Repeat (HR) of SARS-CoV-2 Elicit Robust Protective Immune Responses. *Immunity* **53**, 1315-1330.e1319 (2020).

3 Huang, W. C. *et al.* SARS-CoV-2 RBD Neutralizing Antibody Induction is Enhanced by Particulate Vaccination. *Adv Mater* **32**, e2005637 (2020).

4 Wang, Y. *et al.* Engineering a self-navigated MnARK nanovaccine for inducing potent protective immunity against novel coronavirus. *Nano Today* **38**, 101139 (2021).

5 Volpatti, L. R. *et al.* Polymersomes Decorated with the SARS-CoV-2 Spike Protein Receptor-Binding Domain Elicit Robust Humoral and Cellular Immunity. *ACS Cent Sci* **7**, 1368-1380 (2021).
